# Detection of persistent pestivirus infection in pudú (*Pudu puda*) in a captive population of artiodactyls in Chile

**DOI:** 10.1186/s12917-018-1363-x

**Published:** 2018-02-01

**Authors:** Rodrigo Salgado, Ezequiel Hidalgo-Hermoso, José Pizarro-Lucero

**Affiliations:** 1 Laboratory of Animal Virology, Department of Animal Preventive Medicine, Faculty of Livestock and Veterinary Sciences, University of Chile, Av. Santa Rosa, 11735 Santiago, Chile; 2 Department of Conservation and Research, Buin Zoo Zoological Park, Panamericana Sur Km, 32 Buin, Chile

**Keywords:** Pestivirus, Bovine viral diarrhea virus, Zoo, Virus persistent infection, Pudú

## Abstract

**Background:**

Bovine Viral Diarrhea Virus (BVDV) is the viral agent causing the most important economic losses in livestock throughout the world. Infection of fetuses before their immunological maturity causes the birth of animals persistently infected with BVDV (PI), which are the main source of infection and maintenance of this pathogen in a herd. There is evidence of susceptibility to infection with BVDV in more than 50 species of the order Artiodactyla, and the ability to establish persistent infection in wild cervid species of South America could represent an important risk in control and eradication programs of BVDV in cattle, and a threat to conservation of these wild species. In this study, a serological and virological study was performed to detect BVDV infection in a captive population of non-bovine artiodactyl species in a Chilean zoo with antecedents of abortions whose pathology suggests an infectious etiology.

**Results:**

Detection of neutralizing antibodies against BVDV was performed in 112 artiodactyl animals from a zoo in Chile. Three alpacas (*Vicugna pacos*), one guanaco (*Lama guanicoe*) and seven pudús (*Pudu puda*) resulted seropositive, and the only seronegative pudú was suspected to be persistently infected with BVDV. Then two blood samples nine months apart were analyzed by a viral neutralization test and RT-PCR. Non-cytopathogenic BVDVs were isolated in both samples. A phylogenetic analysis showed that the virus was highly related to BVDV-1b strains circulating among Chilean cattle.

**Conclusions:**

This is the first report of a South American deer persistently infected with Bovine Viral Diarrhea Virus. Further studies are needed to determine the possible role of BVDV as a pathogen in pudús and as a threat to their conservation.

## Background

BVDV is one of the most important infectious agent in livestock industry because it causes high economic impact, affecting reproduction and facilitating secondary infections [1]. BVDV infects the fetus of a pregnant cow with multiple consequences such as abortion, mummification, being stillborn or born weak. If cattle are infected in early pregnancy with a non-cytopathogenic strain, the virus induces immunotolerance in the fetus. These animals are born persistently infected (PI) and continually shed large amounts of the virus into the environment, representing the main source of infection in a herd [2].

BVDV is not limited to cattle; there is evidence of susceptibility to infection in more than 50 species of the order *Artiodactyla* [3]. In addition to cattle, persistent infection has been identified in sheep (*Ovis orientalis aries*), goat (*Capra aegagrus hircus*), swine (*Sus scrofa domestica*), alpaca (*Vicugna pacos*), eland (*Taurotragus oryx*), Java mouse deer (*Tragulus javanicus*), mule deer (*Odocoileus hemionus*), white-tailed deer (*Odocoileus virginianus*) and mountain goat (*Oreamnos americanus*) [4–12]. The ability to establish persistent infection in other animal species emphasizes the importance of surveillance in wild species, which may act as reservoirs, hindering control and eradication programs of BVDV within a country.

Zoos keep a large number of domestic and wild species susceptible to BVDV in high proximity or contact between them. Cases of illness or persistent infection with BVDV have been reported in some animals in zoos [7, 8, 13, 14].

In this study, a serological and virological analysis was performed in a captive population of non-bovine artiodactyl species, with antecedents of abortions whose pathology suggests an infectious etiology, in order to detect BVDV infection in the Zoo.

## Methods

### Serological analysis

#### Animals and sampling

Two samplings were performed during annual preventive procedures in a zoo located in Buin, Región Metropolitana, Chile (Buin-Zoo). The first sampling was between July 2011 and January 2012 (S1) and the second was between July 2012 and January 2013 (S2). Blood samples were collected from 112 individuals comprising 13 species of Order *Artiodactyla* (Table 1). Animal entry and exit occurred during inter-sampling period, however, most animals were sampled in both serological surveys. No animals were previously vaccinated against BVDV. Blood samples were centrifuged at 3500 g for 5 min before serum separation. Sera were stored at − 20 °C until analysis.

**Table 1:** Species and total of sampled animals for serological analysis

Species	Total sampled
Thomson’s gazelle (*Eudorcas thomsonii*)	17
Sitatunga (*Tragelaphus spekii*)	1
Nyala (*Tragelaphus angasii*)	3
Mouflon (*Ovis orientalis orientalis*)	24
Fallow deer (*Dama dama*)	12
Red deer (*Cervus elaphus*)	5
Pudú (*Pudu puda*)	8
Bactrian camel (*Camelus bactrianus*)	2
Guanaco (*Lama guanicoe*)	3
Llama (*Lama glama*)	5
Alpaca (*Vicugna pacos*)	17
Wild boar (*Sus scrofa*)	12
Giraffe (*Giraffa camelopardalis*)	3

#### BVDV antibody detection

Neutralizing antibodies against BVDV were detected by the Virus Neutralization Test (VNT) as described by OIE [15]. In brief, sera were inactivated at 56 °C for 30 min and then serially diluted twofold in minimum essential medium supplemented with equine serum in a 96-well microplate using the MDBK cell line (ATCC CCL-22), determined to be free of BVDV, and 100 tissue culture infective dose 50% (TCID_50_) of the NADL strain of BVDV-1. Bovine sera with and without antibodies against BVDV were used as positive and negative controls, respectively, and titration of infective doses of virus was performed. Microplates were incubated in a 5% CO_2_ atmosphere at 37 °C and cells were examined microscopically at 72 h for cytopathic effect detection. The absence of cytopathic effect was considered positive for the presence of antibodies. The neutralizing antibody titer corresponded to the reciprocal of the highest dilution of serum that neutralized the virus in 50% of the wells. Titers ≥1:4 were considered positive [16]. Due to cross reactivity with other viruses antigenically similar to BVDV, the results are expressed as antibody titers against pestivirus. The MDBK cell line was provided by Dr. Sagar Goyal (University of Minnesota, USA).

### PI animal identification

#### Suspicious PI animal sampling

Since the standard for the diagnosis of BVDV PI animals is the absence of antibodies and presence of the virus in two blood samples collected at least two weeks apart [5], the only pudú that had no neutralizing antibodies against the NADL strain of BVDV in both serological surveys (S1 and S2; see Results) was suspected to be a BVDV PI animal. To confirm this status, two blood samples, nine months apart (January and October 2013), were collected from this individual. From the first sample (SP1) only serum was extracted, while serum and peripheral blood mononuclear cells were extracted from the second sample (SP2).

#### Serological and virological analysis

Neutralizing antibodies against BVDV were detected by VNT in serum from SP1 and SP2 as described above, using the NADL strain of BVDV-1. Serum from SP1 and SP2 was also inoculated onto BVDV-free Madin-Darby Bovine Kidney (MDBK) cells, and after three culture passages pestivirus was detected by amplification of the 5’UTR of the pestivirus genome by RT-PCR. Total RNA was extracted from infected cells using Trizol LS (Invitrogen, Carlsbad, USA), according to the manufacturer’s instructions. Amplification of viral RNA was carried out by RT-PCR in a SuperScript III One-Step RT-PCR System with Platinum Taq DNA Polymerase (Invitrogen, Carlsbad, USA), according to the manufacturer’s instructions and under the following conditions: 12.5 μl 2× Reaction Mix, 0.2 mM dNTP, 2 mM MgSO_4_, 10 pmol of each primer 324 and 326 [17], 5 μl heat-denatured RNA (2 min at 94 °C), and 1 μl SuperScript III RT/Platinum Taq Mix. Synthesis of cDNA was performed in a final volume of 25 μl for 30 min at 55 °C. After reverse transcription, the reaction was heated in a thermocycler for 3 min at 94 °C and then subjected to 30 cycles of 94 °C for 30 s, 55 °C for 1 min and 68 °C for 1 min.

After PCR, DNA amplicon purification was carried out using the QIAquick PCR purification kit (Qiagen, Hilden, Germany), and DNA sequencing was performed in an ABI-310 automated sequencer using dideoxy-sequencing chemistry with the Big Dye Terminator Cycle Sequencing with AmpliTaq DNA polymerase (Applied Biosystems, California, USA). Both strands of each PCR product were sequenced in triplicate from amplified products obtained from individual amplifications.

#### Phylogenetic analysis

The genetic typing of viruses was performed using a 229 nucleotide region of the 5’-UTR (nucleotides 142–371 of the NADL genome). Nucleotide sequences of representative isolates of previously identified phylogenetic groups of BVDV were included in the phylogenetic analysis [18–21].

The nucleotide sequences were aligned using the ClustalW program. A matrix of distances was generated using Kimura’s two-parameter model. The phylogenetic analysis was performed using the neighbor-joining method of the MEGA program [22]. The robustness of the phylogenetic analysis and significance of the branching order were determined by a bootstrap analysis carried out on 1000 replicates.

## Results

Of the 112 animals sampled, 9.82% were positive for antibody to pestivirus; they included alpaca, guanaco and pudú (Table 2).

**Table 2:** Antibody titers for pestivirus in pudús, alpacas and guanaco at the two samplings (S1 and S2)

Species and ID	Antibody titers	
	S1	S2
Guanaco	< 4	64
Alpaca 1	< 4	128
Alpaca 2	< 4	32
Alpaca 3	NS	64
Pudú 1	256	NS^1^
Pudú 2	128	NS^2^
Pudú 3	128	90
Pudú 4	90	512
Pudú 5	64	181
Pudú 6	362	512
Pudú 7	< 4	< 4
Pudú 8	NS^3^	724

NS: Not sampled (not present into the zoo)

NS^1^: Death: 11/18/2011

NS^2^: Death: 01/02/2012

NS^3^: Born: 11/28/2011

There were different results between S1 and S2 within camelid species. The three guanacos evaluated in S1 were seronegative, however, in S2 one of these animals showed antibodies. In alpacas, none of the 13 individuals sampled in S1 showed antibodies against pestiviruses, whereas in S2 three of 14 alpacas were positive. This group of 14 individuals corresponded to 10 animals present in the previous sampling and 4 new alpacas, of which two and one were seropositive, respectively.

**Fig. 1:**
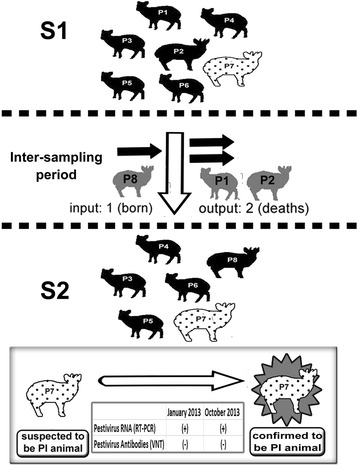
Scheme of serology results of the captive group of pudús at the two samplings (S1 and S2), that led to the suspicion of PI status in one individual

Six of the seven pudús evaluated in S1 were seropositive, while in S2, of six animals evaluated, five that remained from the previous sampling and one new pudú born in the zoo (Pudú 8) were positive to antibodies against pestivirus. The same pudú which was seronegative in S1 was also in S2 (Pudú 7), which made him suspect of being a PI animal (Fig. 1). The two blood samples from the seronegative pudú, obtained nine months apart (January and October 2013), were negative for antibodies against pestivirus but positive for the presence of pestivirus RNA. Virus isolation in MDBK cells indicated that both viruses were non-cytopathogenic strains because no cytopathic effect was observed in culture cells. The PI animal showed no physiological evidence or clinical signs of disease during the study.

**Fig. 2:**
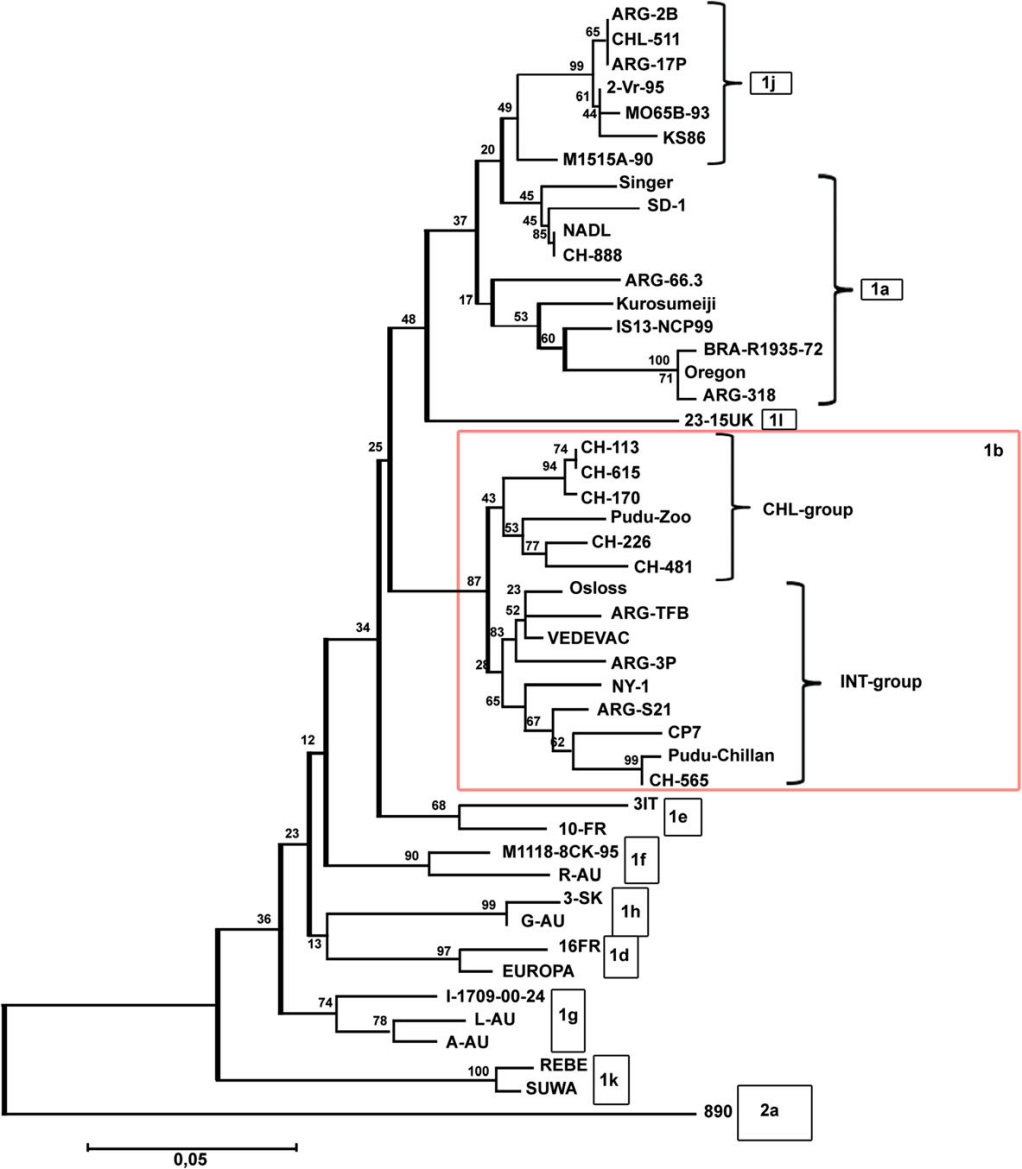
Phylogenetic tree of the 5′ untranslated region sequences from the Pudú/Zoo isolate, Chilean BVDV isolates and other BVDV isolates representative of the major BVDV genotypes and subgenotypes. **BVDV-1a**: Singer (accession number L32875); SD-1 (M96751); NADL (M31182); CH888 (AY671977); ARG/66.3 (AF244967); IS13 NCP/99 (AB042702); Kurosumeiji (AB042655); BRA/R1935/72 (U94916); Oregon (AF091605); ARG/318 (AF244958). **BVDV-1-b**: CH113 (AY671978); CH615 (AY671982); CH170 (AY671979); Pudú-Zoo (KX023408) CH226 (AY671980); CH481 (AF356503); ARG/3P (AF244968); VEDEVAC (AJ585412); Osloss (M96687); ARG/TFB (AF244971); NY-1 (L32879); ARG/S21 (AF244963); CP7 (U63479); Pudú-Chillán (AY679726); CH565 (AY671981). **BVDV-1d**: 16FR (AF298056); EUROPA (AB000898). **BVDV-1e**: 3-IT (AF298062); 10-FR (AF298054). **BVDV-1f**: M1118-8CK/95 (U97421); R-AU (AF298071. **BVDV-1 g**: I-1709/00–24 (AY323877); L-AU (AF298069); A-AU (AF298064). **BVDV-1 h**: 3-SK (AF298068); G-AU (AF298066). **BVDV-1i**: 23/15UK (AF298059). **BVDV-1j**: ARG/2B (AF244957); CHL/511 (AF356504); ARG/17P (AF244954); 2/Vr/95 (AJ293594); M065B/93 (U97409); KS86 (AB078950); M1515A/90 (U97429). **BVDV-1 k**: REBE (AF299317); SUWA (AF117699). **BVDV-2a**: 890 (U18059)

Nucleotide identities among the nucleotide sequence of the two viruses showed 100% identity, confirming that it was the same virus. The nucleotide sequence of this “Pudú-Zoo” isolate was submitted to GenBank (accession number KX023408). Phylogenetic analysis showed that the nucleotide sequence of this isolate belonged to a pestivirus closely related to the BVDV subgenotype 1b circulating among Chilean cattle. The percentage of identity between the nucleotide sequence of the Pudú-Zoo isolate and the other BVDV-1b isolates analyzed ranged from 93 to 96%. The highest genomic identity of the nucleotide sequence (96%) of the Pudú/Zoo isolate was seen with the Chilean cattle isolates CHL-113, CHL-170, and CHL-615 (CHL-group); the lowest genomic identity (93%) was seen with cattle isolates NY-1, CP7 and CHL-565(INT-group) (Fig. 2).

## Discussion

The detection of identical pestivirus in two blood samples taken more than three weeks apart in the absence of antibodies against the virus showed a persistent infection of pestivirus in a captive pudú (*Pudu puda*) in Chile. This is the first report of PI in a deer endemic to South America. This study also reports the first evidence of pestivirus infection in a captive population of wild artiodactyls of South America.

All the animals that resulted positive for antibodies against pestivirus were more than 10 months old at the time of first sampling and were kept in contiguous cages, where direct contact between alpacas and guanaco was possible, while the pudús were housed a few meters apart from the South American camelids. Some animals showed seroconversion in the second sampling, showing the transmission of the virus within the zoo. The most likely pestivirus infecting these animals was BVDV because of the presence of a PI pudú and because BVDV is the only pestivirus reported in Chile, although another pestivirus is not discounted. Even though the main route of transmission of pestivirus is direct contact, the presence of PI animals also facilitates indirect transmission, which can occur through fomites, mechanical vectors or aerosols for a limited time and over short distances [23–25], which may explain the infection of alpacas and guanacos from the pudús; however, other routes of infection for these groups of south American camelids cannot be discounted. The origin of the virus in pudús is also unknown. The PI pudú was born in the zoo, but the identity of his mother was not recorded. Of the six pudús that were seropositive in S1, one was a male born in the zoo and the remaining five came from a captive center in southern Chile, including one female who was pregnant at the time of entering the zoo (Pudú 3). The infection of the mother and the generation of the PI specimen thus may have occurred within the zoo or previously in the other captive center in southern Chile.

Infection with pestiviruses had not been reported in guanacos until now, while in alpacas the prevalence of infection has been reported between 0.9–11.5%, except one case with a herd that had a PI alpaca, where 85% of the animals were seropositive (17 of 20) [5]. Although the proportion of seropositive animals observed in the camelids of our study is lower, the presence of a PI animal among them cannot be discounted. Virus detection in seronegative individuals will be necessary, especially considering that all cases of PI alpacas reported have been by infection with the BVDV-1b subgenotype, which was the subgenotype isolated from the PI pudú. No clinical signs consistent with a pestivirus infection were recorded in the zoo camelids during the inter-sampling period, although they may have gone unnoticed. Acute BVDV infection in South American camelids is almost undetectable, except for vague signs of disease such as anorexia and lethargy [26].

Including our results, antibody titers against pestivirus following natural infection in alpacas ranged from 20 to 480 [26]. However, real titers are uncertain due to possible differences between the infecting virus and the strain used in the diagnostic technique [27].

Neutralizing antibodies against pestivirus in pudús showed an increase at the second sampling, suggestive of reinfection. Titers between sampling periods are comparable because all samples were analyzed simultaneously.

The only previous report of BVDV infection in pudús is from Pizarro-Lucero et al. (2005) [28], who isolated an non-cytopathogenic strain from a free-ranging pudú found dead in southern Chile. This animal had neutralizing antibodies against pestivirus and ulcerative lesions in the nose, mouth, gingival mucosa, interdigital spaces and esophagus, very similar to lesions observed in mucosal disease in cattle. Nevertheless, no cytopathic strain was isolated to associate it with this pathological condition. Pudús did not show clinical signs related to BVDV infection during our study. However, the population of pudús had a history of several deaths by unknown causes. Three pudús included in this study died, two before (Pudú 1, with necropsy findings suggesting infectious disease but no ancillary test to confirm it, and Pudú 2 with necropsy findings not suggesting infectious disease) and one after S2 (Pudú 8 with necropsy findings not suggesting infectious disease). In addition, pudús showed abortions with congenital malformations suggestive of BVDV infection.

Both the pestivirus reported in this study (Pudú-Zoo isolate) and by Pizarro-Lucero et al. (2005) (Pudú-Chillan isolate) were of the BVDV-1b subgenotype. The BVDV-1b subgenotype is one of the most prevalent subgenotypes in cattle in Chile. It is divided into two groups: one group containing only viruses isolated from Chilean cattle (CHL group) and the other group containing viruses isolated from cattle of Chile and other countries (INT group) [29]. Interestingly, the nucleotide sequence from the Pudú-Chillan isolate belongs to the INT group, and the nucleotide sequence from the Pudú-Zoo isolate belongs to the CHL group (Fig. 2). These results suggest that viruses that belong to the BVDV-1b subgenotype have the ability to infect pudús, and transmission of BVDV from cattle to pudú or vice versa could be occurring.

Additional studies should be performed to learn the effects of BVDV infection on pudú health. This virus has shown evidence of causing reproductive disease in white-tailed deer after experimental inoculation and is considered an important emerging disease in the South American camelid industry [30–32]. BVDV has been identified recently as an etiological agent of abortion in sheep flocks, suggesting high potentiality as a threat for new domestic and wild species [33].

The pudú was recently taken off the International Union of Conservation on Nature (IUCN) red list of threatened species; however, enormous fires occurred lately in much of its distribution range in Chile, with consequent mortality and loss of habitat that may change its vulnerability and conservation status [34]. The threat of diseases increases as species move toward extinction [35] and infectious agents that potentially affect individuals and populations through their impact on reproduction, i.e. BVDV and others, could play a role as a threat to conservation of this endemic species of South America [36, 37].

## Conclusions

This study showed for the first time a persistent infection of pestivirus in pudú (*Pudu puda*), a wild deer endemic to South America. The results add a new wildlife species to the list of those with persistent infections of BVDV and a new case report within a zoo.

The proper detection of pathogens that cause persistent infection such as BVDV is also very important for the health of ecosystems, particularly in South American zoos, many of which act as rescue centers for injured or confiscated animals, which are later released to their natural environment.

A PI animal represents a high risk of illness or generation of new PIs for the rest of the captive population in any animal breeding or exhibition center, and the usual management of these animals is their elimination by euthanasia, emphasizing the threat of BVDV for captive endangered species.

Further studies are needed to determine the level of BVDV infection of wild populations of pudús present in southern Chile and the epidemiology of the virus, considering livestock and other susceptible species in the region such as sheep, huemuls (*Hippocamelus bisulcus*) and red deer (*Cervus elaphus*).

## Abbreviations

5’UTR: 5’ Untranslated Region
ATCC: American Type Culture Collection
BVDV: Bovine Viral Diarrhea Virus
BVDV-1b: Bovine Viral Diarrhea Virus subgenotype 1b
MDBK: Madin-Darby Bovine Kidney cell
OIE: World Organisation for Animal Health
PCR: Polymerase Chain Reaction
PI: Persistently Infected
RT-PCR: Reverse Transcription Polymerase Chain Reaction
S1: First Sampling
S2: Second Sampling
TCID_50_: Tissue Culture Infective Dose 50%
VNT: Virus Neutralization Test
